# The Norfolk and Norwich Hospital

**Published:** 1911-02-04

**Authors:** Frank G. Hazell

**Affiliations:** Secretary. Norfolk and Norwich Hospital, Norwich


### INSTITUTIONAL LETTER-BOX.

THE NORFOLK AND NORWICH HOSPITAL.

*To the Editor of* The Hospital.

Dear Sir,—My attention has been drawn to the sixth paragraph under the heading of "Jottings" on p. 517 of your issue dated January 21, 1911.

The paragraph reads as follows: "The sum of £9,681 has been subscribed for the Norfolk memorials to King Edward. Of this sum £329 has been devoted to special purposes, including a bronze bust of the late King at the Norfolk and Norwich Hospital; and the balance, less expenses, is to be applied to the endowment of the Norfolk and Norwich Hospital extension buildings."

As it would appear from this statement that this hospital has benefited by some £9,200, I should be much obliged if you would kindly insert the following statement:

The sum of £9,681 has been subscribed to the Norfolk Memorials to King Edward. Of this sum £4,301 has been allocated to the various hospitals, etc., throughout the county, expenses amount to £175, and £150 has been set aside for the purchase of a bronze bust of King Edward.

The balance of the fund, amounting to about £5,055, is to be applied to the endowment of the King Edward VII. Wards of the Norfolk and Norwich Hospital.

Frank G. Hazell, Secretary.

Norfolk and Norwich Hospital, Norwich,
January 23.

